# Supplementary figures and images for: Aerosol tracer testing in Boeing 767 and 777 aircraft to simulate exposure potential of infectious aerosol such as SARS-CoV-2
Source: PLoS One. 2021 Dec 1;16(12):e0246916. doi: 10.1371/journal.pone.0246916 (PMC8635387; doi:10.1371/journal.pone.0246916)

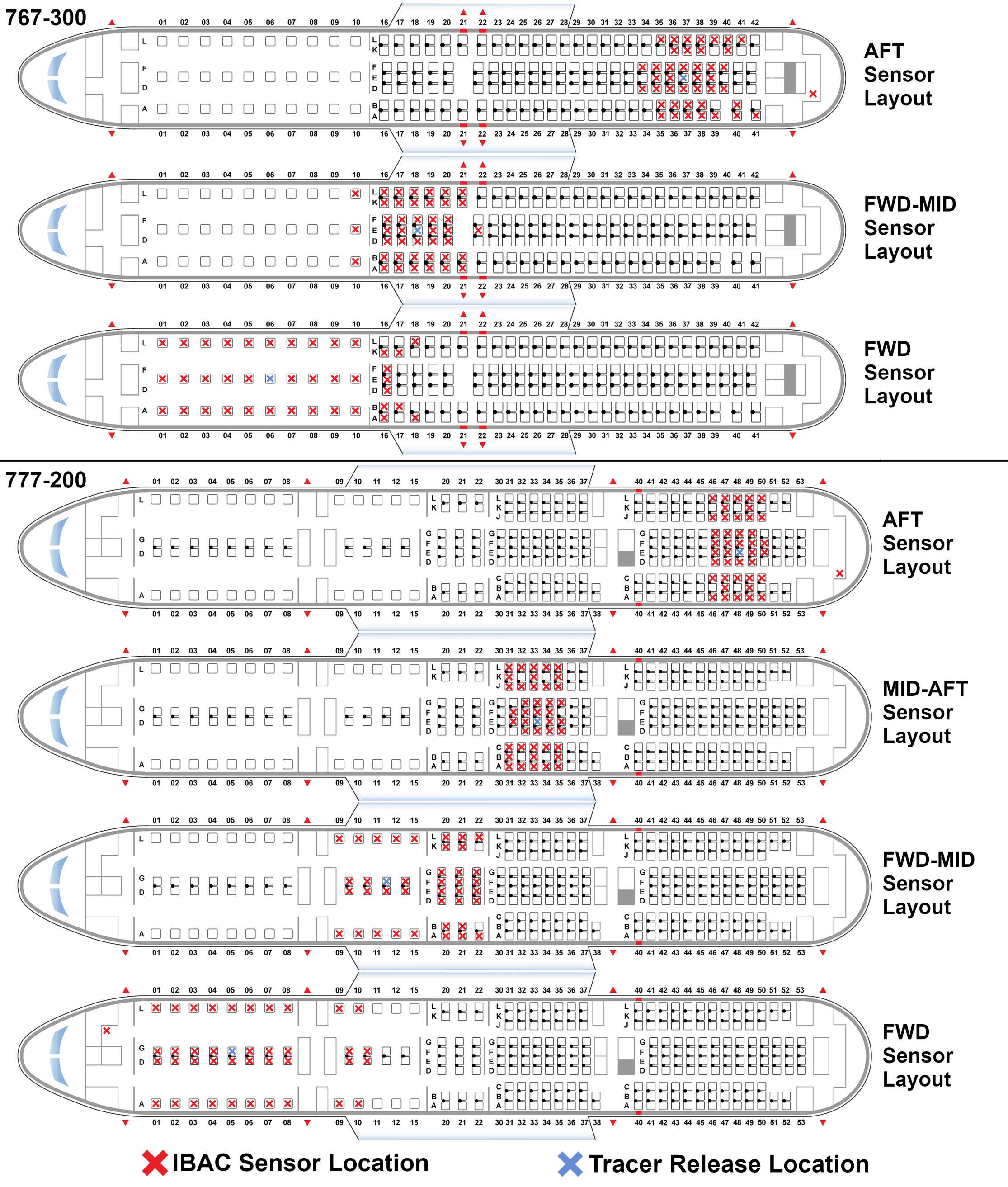

Supplement: S1 Fig — IBAC sensor layouts for each airframe and section tested. A) 767–300 sections and seats B) 777–200 sensors and seats. A single release seat is shown, but releases were done in multiple seats within a given row. (TIF) [file pone.0246916.s001.tif]

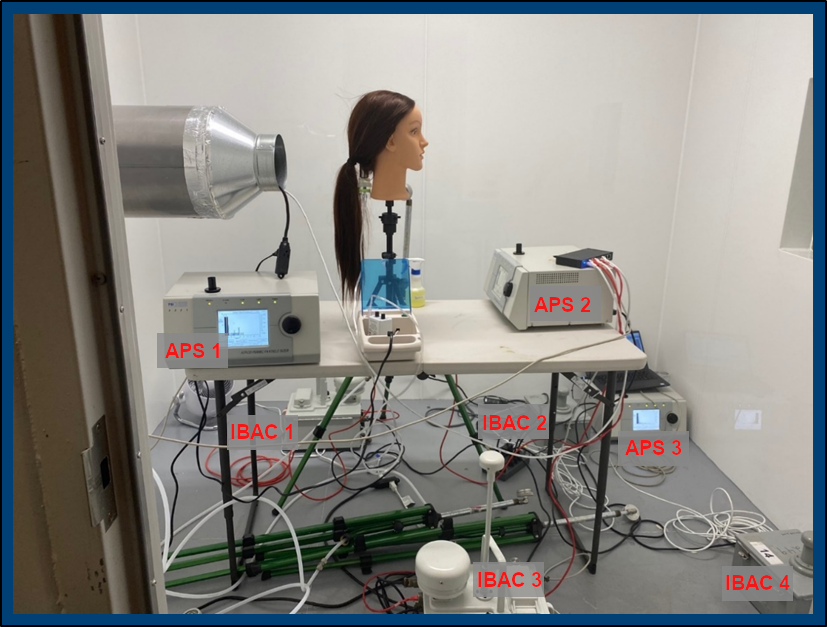

Supplement: S2 Fig — Chamber testing using a mannequin, three APS particle sizers, and four IBACs. (TIF) [file pone.0246916.s002.tif]

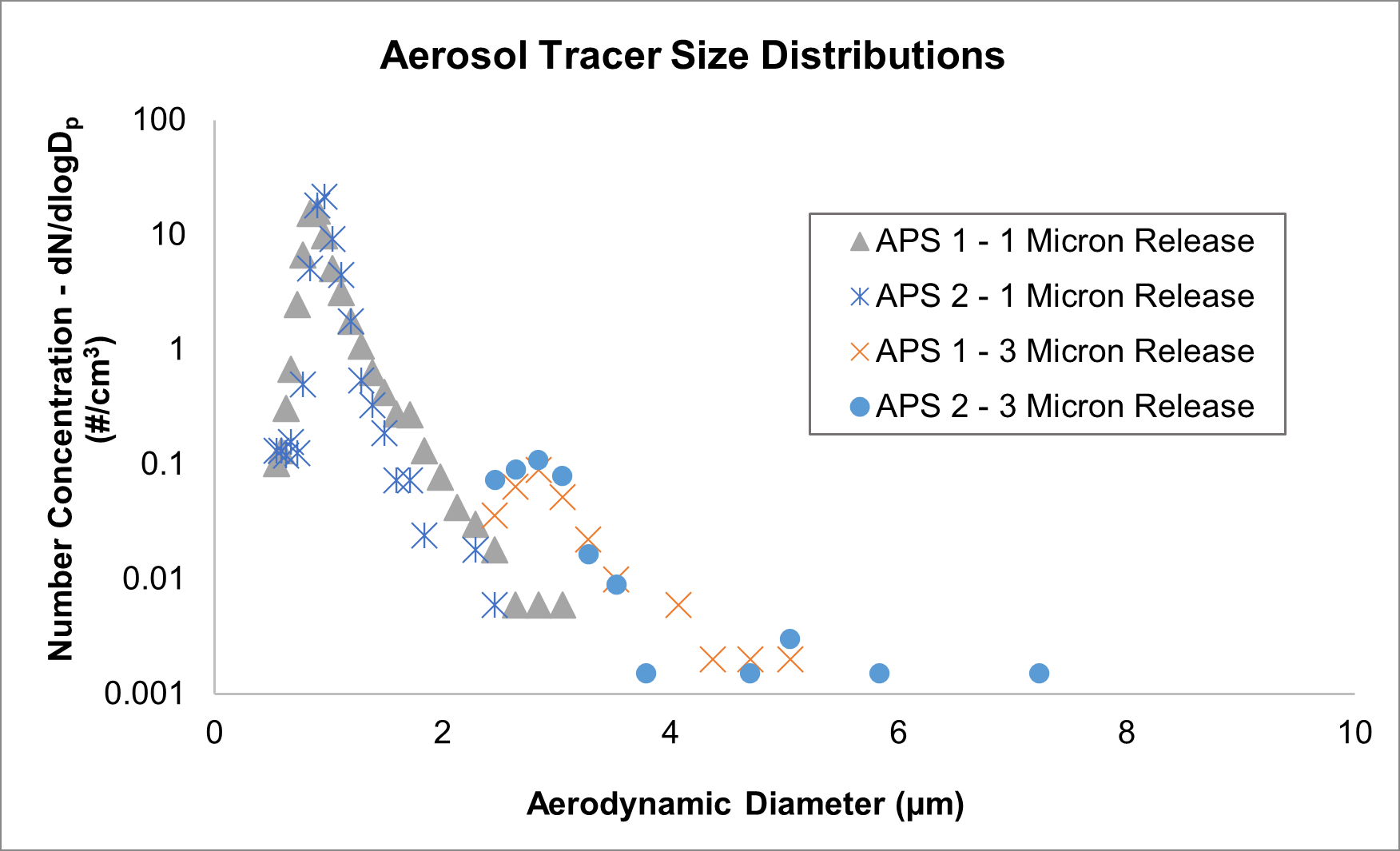

Supplement: S3 Fig — The aerodynamic size distribution for each tracer size normalized for width of size bin (dN/dlogDp). (TIF) [file pone.0246916.s003.tif]

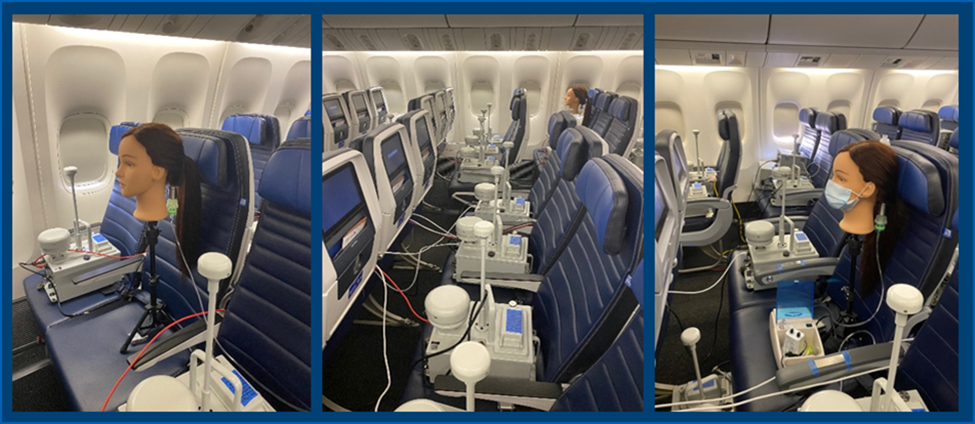

Supplement: S4 Fig — Visualization of mannequin and instruments on a Boeing 767, with and without a mask. (TIF) [file pone.0246916.s004.tif]

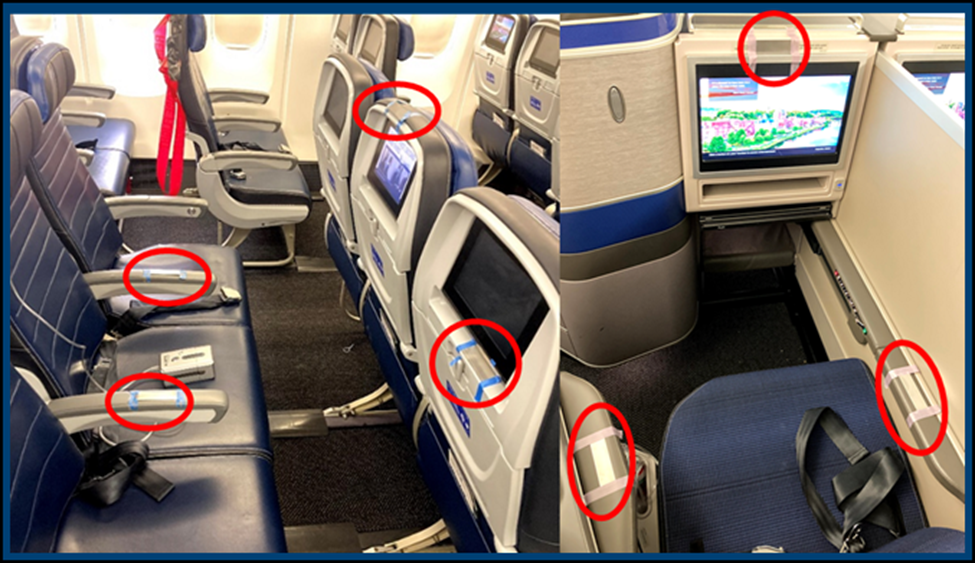

Supplement: S5 Fig — Coupons and locations highlighted in red. Left: Economy seat. Right: First class seat. (TIF) [file pone.0246916.s005.tif]
