# Supplementary material for: Aerosol tracer testing in Boeing 767 and 777 aircraft to simulate exposure potential of infectious aerosol such as SARS-CoV-2
Source: PLoS One. 2021 Dec 1;16(12):e0246916. doi: 10.1371/journal.pone.0246916 (PMC8635387; doi:10.1371/journal.pone.0246916)
Supplement: S1 Table — Hangar testing for the Boeing 777–200 on August 24, 2020. (DOCX) [file pone.0246916.s007.docx]

| **24-Aug-2020** | | **777 Hangar Testing** | | |
| --- | --- | --- | --- | --- |
| **Test** | **Airframe Section** | **Row/Seat** | **Gaspers** | **Mannequin Mask** |
| Test 1 | AFT | 47A | OFF | OFF |
| Test 2 | AFT | 47A | OFF | OFF |
| Test 3 | AFT | 47B | OFF | OFF |
| Test 4 | AFT | 47B | OFF | OFF |
| Test 5 | AFT | 47C | OFF | OFF |
| Test 6 | AFT | 47C | OFF | OFF |
| Test 7 | AFT | 47D | OFF | OFF |
| Test 8 | AFT | 47D | OFF | OFF |
| Test 9 | AFT | 47E | OFF | OFF |
| Test 10 | AFT | 47E | OFF | OFF |
| Test 11 | AFT | 47F | OFF | OFF |
| Test 12 | AFT | 47F | OFF | OFF |
| Test 13 | AFT | 47G | OFF | OFF |
| Test 14 | AFT | 47G | OFF | OFF |
| Test 15 | AFT | 47J | OFF | OFF |
| Test 16 | AFT | 47J | OFF | OFF |
| Test 17 | AFT | 47K | OFF | OFF |
| Test 18 | AFT | 47K | OFF | OFF |
| Test 19 | AFT | 47L | OFF | OFF |
| Test 20 | AFT | 47L | OFF | OFF |
| Test 21 | FWD | 5A | OFF | OFF |
| Test 22 | FWD | 5D | OFF | OFF |
| Test 23 | FWD | 5G | OFF | OFF |
| Test 24 | FWD | 5L | OFF | OFF |
| Test 26 | FWD-MID | 11A | OFF | OFF |
| Test 27 | FWD-MID | 11D | OFF | OFF |
| Test 28 | FWD-MID | 11G | OFF | OFF |
| Test 29 | FWD-MID | 11L | OFF | OFF |
| Test 30 | MID-AFT | 33A | OFF | OFF |
| Test 31 | MID-AFT | 33B | OFF | OFF |
| Test 32 | MID-AFT | 33C | OFF | OFF |
| Test 33 | MID-AFT | 33D | OFF | OFF |
| Test 34 | MID-AFT | 33E | OFF | OFF |
| Test 35 | MID-AFT | 33F | OFF | OFF |
| Test 36 | MID-AFT | 33G | OFF | OFF |
| Test 37 | MID-AFT | 33J | OFF | OFF |
| Test 38 | MID-AFT | 33K | OFF | OFF |
| Test 39 | MID-AFT | 33L | OFF | OFF |

**S1 Table.** **Boeing 777-200 Test Conditions and Timeline for Hangar.** Hangar testing for the Boeing 777-200 on August 24, 2020.
