# Supplementary material for: Aerosol tracer testing in Boeing 767 and 777 aircraft to simulate exposure potential of infectious aerosol such as SARS-CoV-2
Source: PLoS One. 2021 Dec 1;16(12):e0246916. doi: 10.1371/journal.pone.0246916 (PMC8635387; doi:10.1371/journal.pone.0246916)
Supplement: S2 Table — Jetway testing for the Boeing 777–200 on August 25, 2020. (DOCX) [file pone.0246916.s008.docx]

| **25-Aug-2020** | | **777 Jetway Testing** | | | | |
| --- | --- | --- | --- | --- | --- | --- |
| **Test** | **AirFrame Section** | **Row/Seat** | **Gaspers** | **Mannequin Mask** | **ECS Conditions** | **Heat Blanket** |
| Test 1 | MID-AFT | 33E | ON | OFF | Ground Air On/Recirc Off | ON |
| Test 2 | MID-AFT | 33E | OFF | OFF | Ground Air On/Recirc Off | ON |
| Test 3 | MID-AFT | 33E | ON | OFF | PACS on/Recirc On | ON |
| Test 4 | MID-AFT | 33E | ON | OFF | PACS on/Recirc On | OFF |
| Test 5 | MID-AFT | 33E | OFF | OFF | PACS on/Recirc On | OFF |
| Test 6 | MID-AFT | 33E | ON | ON | PACS on/Recirc On | OFF |
| Test 7 | MID-AFT | 33E | ON | ON | PACS on/Recirc On | OFF |
| Test 8 | MID-AFT | 33E | ON | OFF | PACS on/Recirc On | OFF |
| Test 9 | FWD-MID | 11G | OFF | OFF | PACS on/Recirc On | OFF |
| Test 10 | FWD-MID | 11G | ON | OFF | PACS on/Recirc On | OFF |
| Test 11 | FWD-MID | 11G | ON | OFF | PACS on/Recirc On | OFF |
| Test 12 | FWD-MID | 11G | ON | ON | PACS on/Recirc On | OFF |
| Test 13 | FWD-MID | 11G | ON | ON | PACS on/Recirc On | OFF |
| Test 14 | FWD-MID | 11G | ON | ON | PACS on/Recirc On | OFF |
| Test 15 | FWD-MID | 11G | ON | OFF | PACS on/Recirc On | OFF |
| Test 16 | FWD-MID | 11G | ON | OFF | PACS on/Recirc On | OFF |
| Test 17 | FWD-MID | 11G | ON | OFF | PACS on/Recirc On | OFF |
| Test 18 | FWD-MID | 11G | ON | OFF | PACS on/Recirc On | OFF |
| Test 19 | FWD-MID | 11G | ON | ON | PACS on/Recirc On | OFF |
| Test 20 | FWD-MID | 11G | ON | OFF | PACS on/Recirc On | OFF |
| Test 21 | FWD-MID | 11G | ON | ON | PACS on/Recirc On | OFF |
| Test 22 | FWD-MID | 11G | ON | OFF | PACS on/Recirc On | OFF |
| Test 23 | FWD-MID | 11G | ON | OFF | PACS on/Recirc On | OFF |
| Test 24 | AFT | 47E | OFF | OFF | PACS on/Recirc On | OFF |
| Test 25 | AFT | 47E | OFF | OFF | PACS on/Recirc On | OFF |

**S2 Table.** **Boeing 777-200 Test Conditions and Timeline for Jetway.** Jetway testing for the Boeing 777-200 on August 25, 2020.
