# Supplementary material for: Aerosol tracer testing in Boeing 767 and 777 aircraft to simulate exposure potential of infectious aerosol such as SARS-CoV-2
Source: PLoS One. 2021 Dec 1;16(12):e0246916. doi: 10.1371/journal.pone.0246916 (PMC8635387; doi:10.1371/journal.pone.0246916)
Supplement: S4 Table — Inflight testing day 2 for the Boeing 777–200 on August 27, 2020. (DOCX) [file pone.0246916.s010.docx]

| **27-Aug-2020** | | **777 Inflight Testing 2** | | |
| --- | --- | --- | --- | --- |
| **Test** | **Airframe Section** | **Row/Seat** | **Gaspers** | **Mannequin Mask** |
| Test 34 | FWD-MID | 11A | OFF | OFF |
| Test 35 | FWD-MID | 11A | OFF | OFF |
| Test 36 | FWD-MID | 11A | OFF | OFF |
| Test 37 | FWD-MID | 11A | OFF | ON |
| Test 38 | FWD-MID | 11A | OFF | ON |
| Test 39 | FWD-MID | 11A | OFF | ON |
| Test 40 | FWD-MID | 11G | OFF | OFF |
| Test 41 | FWD-MID | 11G | OFF | OFF |
| Test 42 | FWD-MID | 11G | OFF | OFF |
| Test 43 | FWD-MID | 11G | OFF | ON |
| Test 44 | FWD-MID | 11G | OFF | ON |
| Test 45 | FWD-MID | 11G | OFF | ON |
| Test 46 | FWD-MID | 11L | OFF | OFF |
| Test 47 | FWD-MID | 11L | OFF | OFF |
| Test 48 | FWD-MID | 11L | OFF | OFF |
| Test 49 | FWD-MID | 11L | OFF | ON |
| Test 50 | FWD-MID | 11L | OFF | ON |
| Test 51 | FWD-MID | 11L | OFF | ON |
| Test 52 | FWD | 5A | OFF | OFF |
| Test 53 | FWD | 5A | OFF | OFF |
| Test 54 | FWD | 5A | OFF | OFF |
| Test 55 | FWD | 5A | OFF | ON |
| Test 56 | FWD | 5A | OFF | ON |
| Test 57 | FWD | 5A | OFF | ON |
| Test 59 | FWD | 5G | OFF | OFF |
| Test 60 | FWD | 5G | OFF | OFF |
| Test 61 | FWD | 5G | OFF | OFF |
| Test 62 | FWD | 5L | OFF | OFF |
| Test 63 | FWD | 5L | OFF | OFF |
| Test 64 | FWD | 5L | OFF | OFF |

**S4 Table.** **Boeing 777-200 Test Conditions and Timeline for Second Day of Inflight Testing.** Inflight testing day 2 for the Boeing 777-200 on August 27, 2020.
