# Supplementary material for: Aerosol tracer testing in Boeing 767 and 777 aircraft to simulate exposure potential of infectious aerosol such as SARS-CoV-2
Source: PLoS One. 2021 Dec 1;16(12):e0246916. doi: 10.1371/journal.pone.0246916 (PMC8635387; doi:10.1371/journal.pone.0246916)
Supplement: S5 Table — Hangar testing for the Boeing 767–300 on August 28, 2020. (DOCX) [file pone.0246916.s011.docx]

| **28-Aug-2020** | | **767 Hangar Testing** | | |
| --- | --- | --- | --- | --- |
| **Test** | **Section** | **Row/Seat** | **Gaspers** | **Mannequin Mask** |
| Test 1 | AFT | 37A | OFF | OFF |
| Test 2 | AFT | 37A | OFF | OFF |
| Test 3 | AFT | 37A | OFF | OFF |
| Test 4 | AFT | 37B | OFF | OFF |
| Test 5 | AFT | 37B | OFF | OFF |
| Test 6 | AFT | 37B | OFF | OFF |
| Test 7 | AFT | 37D | OFF | OFF |
| Test 8 | AFT | 37D | OFF | OFF |
| Test 9 | AFT | 37D | OFF | OFF |
| Test 10 | AFT | 37E | OFF | OFF |
| Test 11 | AFT | 37E | OFF | OFF |
| Test 12 | AFT | 37E | OFF | OFF |
| Test 13 | AFT | 37F | OFF | OFF |
| Test 14 | AFT | 37F | OFF | OFF |
| Test 15 | AFT | 37F | OFF | OFF |
| Test 16 | AFT | 37K | OFF | OFF |
| Test 17 | AFT | 37K | OFF | OFF |
| Test 18 | AFT | 37K | OFF | OFF |
| Test 19 | AFT | 37L | OFF | OFF |
| Test 20 | AFT | 37L | OFF | OFF |
| Test 21 | AFT | 37L | OFF | OFF |
| Test 22 | FWD | 8B | OFF | OFF |
| Test 23 | FWD | 8B | OFF | OFF |
| Test 24 | FWD | 8B | OFF | OFF |
| Test 26 | FWD | 8D | OFF | OFF |
| Test 27 | FWD | 8D | OFF | OFF |
| Test 28 | FWD | 6D | OFF | OFF |
| Test 29 | FWD | 6D | OFF | OFF |
| Test 30 | FWD | 6D | OFF | OFF |
| Test 31 | FWD | 5L | OFF | OFF |
| Test 32 | FWD | 5L | OFF | OFF |
| Test 33 | FWD | 5L | OFF | OFF |
| Test 34 | FWD-MID | 18A | OFF | OFF |
| Test 35 | FWD-MID | 18A | OFF | OFF |
| Test 36 | FWD-MID | 18A | OFF | OFF |
| Test 37 | FWD-MID | 18B | OFF | OFF |
| Test 38 | FWD-MID | 18B | OFF | OFF |
| Test 39 | FWD-MID | 18B | OFF | OFF |
| Test 40 | FWD-MID | 18D | OFF | OFF |
| Test 41 | FWD-MID | 18D | OFF | OFF |
| Test 42 | FWD-MID | 18D | OFF | OFF |
| Test 43 | FWD-MID | 18E | OFF | OFF |
| Test 44 | FWD-MID | 18E | OFF | OFF |
| Test 45 | FWD-MID | 18E | OFF | OFF |
| Test 46 | FWD-MID | 18F | OFF | OFF |
| Test 47 | FWD-MID | 18F | OFF | OFF |
| Test 48 | FWD-MID | 18F | OFF | OFF |
| Test 49 | FWD-MID | 18K | OFF | OFF |
| Test 50 | FWD-MID | 18K | OFF | OFF |
| Test 51 | FWD-MID | 18K | OFF | OFF |
| Test 52 | FWD-MID | 18L | OFF | OFF |
| Test 53 | FWD-MID | 18L | OFF | OFF |
| Test 54 | FWD-MID | 18L | OFF | OFF |

**S5 Table.** **Boeing 767-300 Test Conditions and Timeline for Hangar.** Hangar testing for the Boeing 767-300 on August 28, 2020.
