# Supplementary material for: Aerosol tracer testing in Boeing 767 and 777 aircraft to simulate exposure potential of infectious aerosol such as SARS-CoV-2
Source: PLoS One. 2021 Dec 1;16(12):e0246916. doi: 10.1371/journal.pone.0246916 (PMC8635387; doi:10.1371/journal.pone.0246916)
Supplement: S6 Table — Jetway testing for the Boeing 767–300 on August 29, 2020. (DOCX) [file pone.0246916.s012.docx]

| **29-Aug-2020** | | **767 Jetway Testing** | | | | |
| --- | --- | --- | --- | --- | --- | --- |
| **Test** | **Section** | **Seat** | **Gaspers** | **Mannequin Mask** | **Test Condition** | **Heat Blanket** |
| Test 1 | FWD-MID | 18E | ON | OFF | Ground Air ON/ Recirc ON | ON |
| Test 2 | FWD-MID | 18E | ON | OFF | Ground Air ON/ Recirc ON | ON |
| Test 3 | FWD-MID | 18E | ON | OFF | Ground Air ON/ Recirc ON | ON |
| Test 4 | FWD-MID | 18E | OFF | OFF | Ground Air ON/ Recirc ON | ON |
| Test 5 | FWD-MID | 18E | OFF | OFF | Ground Air ON/ Recirc ON | ON |
| Test 6 | FWD-MID | 18E | OFF | OFF | Ground Air ON/ Recirc ON | ON |
| Test 7 | FWD-MID | 18E | ON | OFF | PACS ON /Recirc ON | ON |
| Test 8 | FWD-MID | 18E | ON | OFF | PACS ON /Recirc ON | ON |
| Test 9 | FWD-MID | 18E | ON | OFF | PACS ON /Recirc ON | ON |
| Test 10 | FWD-MID | 18E | ON | OFF | PACS ON /Recirc ON | OFF |
| Test 11 | FWD-MID | 18E | ON | OFF | PACS ON /Recirc ON | OFF |
| Test 12 | FWD-MID | 18E | ON | OFF | PACS ON /Recirc ON | OFF |
| Test 13 | FWD-MID | 18E | OFF | OFF | PACS ON /Recirc ON | OFF |
| Test 14 | FWD-MID | 18E | OFF | OFF | PACS ON /Recirc ON | OFF |
| Test 15 | FWD-MID | 18E | OFF | OFF | PACS ON /Recirc ON | OFF |
| Test 16 | FWD-MID | 18E | OFF | OFF | PACS ON /Recirc ON | OFF |
| Test 17 | FWD-MID | 18E | OFF | OFF | PACS ON /Recirc ON | OFF |
| Test 18 | FWD-MID | 18E | OFF | ON | PACS ON /Recirc ON | OFF |
| Test 19 | FWD-MID | 18E | OFF | ON | PACS ON /Recirc ON | OFF |
| Test 20 | FWD-MID | 18E | OFF | ON | PACS ON /Recirc ON | OFF |
| Test 21 | FWD | 6D | ON | OFF | PACS ON /Recirc ON | OFF |
| Test 22 | FWD | 6D | ON | OFF | PACS ON /Recirc ON | OFF |
| Test 23 | FWD | 6D | ON | OFF | PACS ON /Recirc ON | OFF |
| Test 24 | FWD | 6D | ON | OFF | PACS ON /Recirc ON | OFF |
| Test 25 | FWD | 6D | ON | ON | PACS ON /Recirc ON | OFF |
| Test 26 | FWD | 6D | ON | ON | PACS ON /Recirc ON | OFF |
| Test 27 | FWD | 6D | ON | ON | PACS ON /Recirc ON | OFF |
| Test 28 | AFT | 37E | ON | OFF | PACS ON /Recirc ON | OFF |
| Test 29 | AFT | 37E | ON | OFF | PACS ON /Recirc ON | OFF |
| Test 30 | AFT | 37E | ON | OFF | PACS ON /Recirc ON | OFF |
| Test 31 | AFT | 37E | ON | ON | PACS ON /Recirc ON | OFF |
| Test 32 | AFT | 37E | ON | ON | PACS ON /Recirc ON | OFF |
| Test 33 | AFT | 37E | ON | ON | PACS ON /Recirc ON | OFF |

**S6 Table.** **Boeing 767-300 Test Conditions and Timeline for Jetway.** Jetway testing for the Boeing 767-300 on August 29, 2020.
