# Supplementary material for: Aerosol tracer testing in Boeing 767 and 777 aircraft to simulate exposure potential of infectious aerosol such as SARS-CoV-2
Source: PLoS One. 2021 Dec 1;16(12):e0246916. doi: 10.1371/journal.pone.0246916 (PMC8635387; doi:10.1371/journal.pone.0246916)
Supplement: S7 Table — Inflight testing day 1 for the Boeing 767–300 on August 30, 2020. (DOCX) [file pone.0246916.s013.docx]

| **30-Aug-2020** | | **767 Inflight Testing 1** | | | |
| --- | --- | --- | --- | --- | --- |
| **Test** | **Section** | **Row/Seat** | **Gaspers** | **Mannequin Mask** | **Test Condition** |
| Test 1 | AFT | 37B | OFF | OFF | Breathing |
| Test 2 | AFT | 37B | OFF | OFF | Breathing |
| Test 3 | AFT | 37B | OFF | OFF | Breathing |
| Test 4 | AFT | 37B | OFF | ON | Breathing |
| Test 5 | AFT | 37B | OFF | ON | Breathing |
| Test 6 | AFT | 37B | OFF | ON | Breathing |
| Test 7 | AFT | 37E | OFF | OFF | Breathing |
| Test 8 | AFT | 37E | OFF | OFF | Breathing |
| Test 9 | AFT | 37E | OFF | OFF | Breathing |
| Test 10 | AFT | 37E | OFF | ON | Breathing |
| Test 11 | AFT | 37E | OFF | ON | Breathing |
| Test 12 | AFT | 37E | OFF | ON | Breathing |
| Test 13 | AFT | 37E | OFF | OFF | Coughing |
| Test 14 | AFT | 37E | OFF | OFF | Coughing |
| Test 15 | AFT | 37E | OFF | OFF | Coughing |
| Test 16 | AFT | 37E | OFF | ON | Coughing |
| Test 17 | AFT | 37E | OFF | ON | Coughing |
| Test 18 | AFT | 37E | OFF | ON | Coughing |
| Test 19 | AFT | 37E | OFF | OFF | Coughing |
| Test 20 | AFT | 37K | OFF | OFF | Breathing |
| Test 21 | AFT | 37K | OFF | OFF | Breathing |
| Test 22 | AFT | 37K | OFF | OFF | Breathing |
| Test 23 | AFT | 37K | OFF | ON | Breathing |
| Test 24 | AFT | 37K | OFF | ON | Breathing |
| Test 25 | AFT | 37K | OFF | ON | Breathing |
| Test 26 | AFT | 37K | OFF | OFF | Coughing |
| Test 27 | AFT | 37K | OFF | OFF | Coughing |
| Test 28 | AFT | 37K | OFF | OFF | Coughing |
| Test 29 | AFT | 37K | OFF | ON | Coughing |
| Test 30 | AFT | 37K | OFF | ON | Coughing |
| Test 31 | AFT | 37K | OFF | ON | Coughing |
| Test 32 | FWD-MID | 18A | OFF | OFF | Breathing |
| Test 33 | FWD-MID | 18A | OFF | OFF | Breathing |
| Test 34 | FWD-MID | 18A | OFF | OFF | Breathing |
| Test 35 | FWD-MID | 18A | OFF | ON | Breathing |
| Test 36 | FWD-MID | 18A | OFF | ON | Breathing |
| Test 37 | FWD-MID | 18A | OFF | ON | Breathing |
| Test 38 | FWD-MID | 18A | OFF | OFF | Coughing |
| Test 39 | FWD-MID | 18A | OFF | OFF | Coughing |
| Test 40 | FWD-MID | 18A | OFF | OFF | Coughing |
| Test 41 | FWD-MID | 18A | OFF | ON | Coughing |
| Test 42 | FWD-MID | 18A | OFF | ON | Coughing |
| Test 43 | FWD-MID | 18A | OFF | ON | Breathing |
| Test 44 | FWD-MID | 18E | OFF | OFF | Breathing |
| Test 45 | FWD-MID | 18E | OFF | OFF | Breathing |
| Test 46 | FWD-MID | 18E | OFF | OFF | Breathing |
| Test 47 | FWD-MID | 18E | OFF | ON | Breathing |

**S7 Table.** **Boeing 767-300 Test Conditions and Timeline for First Day of Inflight Testing.** Inflight testing day 1 for the Boeing 767-300 on August 30, 2020.
