# Supplementary material for: Aerosol tracer testing in Boeing 767 and 777 aircraft to simulate exposure potential of infectious aerosol such as SARS-CoV-2
Source: PLoS One. 2021 Dec 1;16(12):e0246916. doi: 10.1371/journal.pone.0246916 (PMC8635387; doi:10.1371/journal.pone.0246916)
Supplement: S8 Table — Inflight testing day 2 for the Boeing 767–300 on August 31, 2020. (DOCX) [file pone.0246916.s014.docx]

| **31-Aug-2020** | | **767 Inflight Testing 2** | | | |
| --- | --- | --- | --- | --- | --- |
| **Test** | **Section** | **Row/Seat** | **Gaspers** | **Mannequin Mask** | **Test Condition** |
| Test 48 | FWD-MID | 18E | OFF | ON | Breathing |
| Test 49 | FWD-MID | 18E | OFF | ON | Breathing |
| Test 50 | FWD-MID | 18L | OFF | OFF | Breathing |
| Test 51 | FWD-MID | 18L | OFF | OFF | Breathing |
| Test 52 | FWD-MID | 18L | OFF | OFF | Breathing |
| Test 53 | FWD-MID | 18L | OFF | ON | Breathing |
| Test 54 | FWD-MID | 18L | OFF | ON | Breathing |
| Test 55 | FWD-MID | 18L | OFF | ON | Breathing |
| Test 56 | FWD | 6A | OFF | OFF | Breathing |
| Test 57 | FWD | 6A | OFF | OFF | Breathing |
| Test 58 | FWD | 6A | OFF | OFF | Breathing |
| Test 59 | FWD | 6A | OFF | ON | Breathing |
| Test 60 | FWD | 6A | OFF | ON | Breathing |
| Test 61 | FWD | 6A | OFF | ON | Breathing |
| Test 62 | FWD | 6A | OFF | OFF | Coughing |
| Test 63 | FWD | 6A | OFF | OFF | Coughing |
| Test 64 | FWD | 6A | OFF | OFF | Coughing |
| Test 65 | FWD | 6A | OFF | ON | Coughing |
| Test 66 | FWD | 6A | OFF | ON | Coughing |
| Test 67 | FWD | 6A | OFF | ON | Coughing |
| Test 68 | FWD | 6D | OFF | OFF | Breathing |
| Test 69 | FWD | 6D | OFF | OFF | Breathing |
| Test 70 | FWD | 6D | OFF | OFF | Breathing |
| Test 71 | FWD | 6D | OFF | ON | Breathing |
| Test 72 | FWD | 6D | OFF | ON | Breathing |
| Test 73 | FWD | 6D | OFF | ON | Breathing |
| Test 74 | FWD | 6L | OFF | OFF | Breathing |
| Test 75 | FWD | 6L | OFF | OFF | Breathing |
| Test 76 | FWD | 6L | OFF | OFF | Breathing |
| Test 77 | FWD | 6L | OFF | ON | Breathing |
| Test 78 | FWD | 6L | OFF | ON | Breathing |
| Test 79 | FWD | 6L | OFF | ON | Breathing |
| Test 80 | FWD | 6L | OFF | OFF | Coughing |
| Test 81 | FWD | 6L | OFF | ON | Coughing |
| Test 82 | FWD | 6L | OFF | OFF | Coughing |
| Test 83 | FWD | 6L | OFF | ON | Coughing |
| Test 84 | FWD | 6L | OFF | OFF | Coughing |
| Test 85 | FWD | 6L | OFF | ON | Coughing |

**S8 Table.** **Boeing 767-300 Test Conditions and Timeline for Second Day of Inflight Testing.** Inflight testing day 2 for the Boeing 767-300 on August 31, 2020.
