# Supplementary material for: Aerosol tracer testing in Boeing 767 and 777 aircraft to simulate exposure potential of infectious aerosol such as SARS-CoV-2
Source: PLoS One. 2021 Dec 1;16(12):e0246916. doi: 10.1371/journal.pone.0246916 (PMC8635387; doi:10.1371/journal.pone.0246916)
Supplement: S9 Table — Large confidence intervals (n = 3, 95% CI based on standard error of the mean) reflect the low nucleic-acid signal. (DOCX) [file pone.0246916.s015.docx]

| **Percent of Released Particles in 1 ft^2^ (Surface Sample) or**  **Integrated Collection at a Given Seat (Aerosol)** | | | | | | | |
| --- | --- | --- | --- | --- | --- | --- | --- |
| **Seat** | **Location** | **FWD** | **±95% CI** | **MID-FWD** | **±95% CI** | **MID-AFT** | **±95% CI** |
| 5D | Center Above IFE | 0.001% | 0.002% | 0.001% | 0.003% | 0.000% | 0.000% |
| 5D | Left Arm Rest | 0.001% | 0.001% | 0.001% | 0.002% | 0.000% | 0.000% |
| 5D | Right Arm Rest | 0.003% | 0.007% | 0.001% | 0.002% | 0.000% | 0.000% |
| 11D | Center Above IFE | 0.000% | 0.000% | 0.002% | 0.004% | 0.000% | 0.000% |
| 11D | Left Arm Rest | 0.000% | 0.000% | 0.001% | 0.003% | 0.000% | 0.001% |
| 11D | Right Arm Rest | 0.000% | 0.000% | 0.017% | 0.023% | 0.000% | 0.000% |
| 33D | Center Above IFE | 0.000% | 0.001% | 0.001% | 0.003% | 0.000% | 0.002% |
| 33E | Center Below IFE | 0.000% | 0.000% | 0.001% | 0.002% | 0.001% | 0.002% |
| 33E | Left Arm Rest | 0.000% | 0.001% | 0.002% | 0.004% | 0.018% | 0.060% |
| 33E | Right Arm Rest | 0.000% | 0.000% | 0.001% | 0.002% | 0.001% | 0.002% |
| 47E | Center Below IFE | 0.000% | 0.000% | 0.000% | #DIV/0! | 0.001% | 0.001% |
| 47E | Left Arm Rest | 0.000% | 0.000% | 0.000% | #DIV/0! | 0.000% | 0.000% |
| 47E | Right Arm Rest | 0.000% | 0.000% | 0.000% | #DIV/0! | 0.001% | 0.001% |
| 8D | Aerosol | 0.000% | 0.000% | 0.000% | 0.001% | 0.000% | 0.000% |
| 12D | Aerosol | 0.000% | 0.000% | 0.004% | 0.008% | 0.001% | 0.001% |
| 36E | Aerosol | 0.000% | 0.000% | 0.000% | 0.000% | 0.030% | 0.093% |
| 49D | Aerosol | 0.000% | 0.000% | 0.000% | 0.000% | 0.007% | 0.017% |
| Rear Galley | Aerosol | 0.000% | 0.001% | 0.000% | 0.000% | 0.002% | 0.006% |

**Table S9.** **777-200 DNA-Tagged Tracer Results.** Large confidence intervals (n=3, 95% CI based on standard error of the mean) reflect the low nucleic-acid signal.
