# Supplementary material for: Aerosol tracer testing in Boeing 767 and 777 aircraft to simulate exposure potential of infectious aerosol such as SARS-CoV-2
Source: PLoS One. 2021 Dec 1;16(12):e0246916. doi: 10.1371/journal.pone.0246916 (PMC8635387; doi:10.1371/journal.pone.0246916)
Supplement: S10 Table — Large confidence intervals (n = 3, 95% CI based on standard error of the mean) reflect the low nucleic-acid signal. (DOCX) [file pone.0246916.s016.docx]

|  | |  | **Percent of Released Particles in 1 Ft2 (Surface Sample) or Integrated Collection at a Given Seat (Aerosol)** | | | | | | |
| --- | --- | --- | --- | --- | --- | --- | --- | --- | --- |
| **Seat** | **Location** | | | **FWD** | **±95% CI** | **MID** | **±95% CI** | **AFT** | **±95% CI** |
| 6D | Left Arm Rest | | | 0.001% | 0.002% | 0.002% | 0.003% | 0.000% | 0.000% |
| 6D | Center Above IFE | | | 0.001% | 0.001% | 0.001% | 0.003% | 0.000% | 0.000% |
| 6D | Right Arm Rest | | | 0.003% | 0.009% | 0.003% | 0.008% | 0.002% | 0.008% |
| 6D | Marble Table | | | 0.003% | 0.004% | 0.005% | 0.005% | 0.000% | 0.001% |
| 18E | Left Arm Rest | | | 0.001% | 0.001% | 0.005% | 0.012% | 0.003% | 0.009% |
| 18E | Center Above IFE | | | 0.000% | 0.001% | 0.002% | 0.003% | 0.002% | 0.006% |
| 18E | Right Arm Rest | | | 0.000% | 0.001% | 0.001% | 0.003% | 0.000% | 0.001% |
| 18F | Center Below IFE | | | 0.000% | 0.000% | 0.001% | 0.002% | 0.000% | 0.002% |
| 26E | Tray Table | | | 0.000% | 0.001% | 0.002% | 0.003% | 0.003% | 0.008% |
| 37D | Center Above IFE | | | 0.000% | 0.001% | 0.000% | 0.000% | 0.001% | 0.003% |
| 37E | Left Arm Rest | | | 0.000% | 0.001% | 0.001% | 0.002% | 0.004% | 0.005% |
| 37E | Center Below IFE | | | 0.000% | 0.001% | 0.002% | 0.007% | 0.002% | 0.006% |
| 37E | Right Arm Rest | | | 0.000% | 0.001% | 0.001% | 0.002% | 0.001% | 0.001% |
| 5F | Aerosol | | | 0.004% | 0.012% | 0.000% | 0.000% | 0.000% | 0.000% |
| 22F | Aerosol | | | 0.000% | 0.000% | 0.000% | 0.001% | 0.000% | 0.001% |
| 31D | Aerosol | | | 0.000% | 0.000% | 0.004% | 0.008% | 0.001% | 0.004% |
| 40F | Aerosol | | | 0.000% | 0.000% | 0.000% | 0.000% | 0.012% | 0.016% |
| Rear Galley | Aerosol | | | 0.000% | 0.000% | 0.000% | 0.000% | 0.014% | 0.001% |

**Table S10.** **767-300 DNA-Tagged Tracer Results.** Large confidence intervals (n=3, 95% CI based on standard error of the mean) reflect the low nucleic-acid signal.
