# Supplementary material for: Aerosol tracer testing in Boeing 767 and 777 aircraft to simulate exposure potential of infectious aerosol such as SARS-CoV-2
Source: PLoS One. 2021 Dec 1;16(12):e0246916. doi: 10.1371/journal.pone.0246916 (PMC8635387; doi:10.1371/journal.pone.0246916)
Supplement: Supplementary file 12 [file pone.0246916.s006.docx]

8/28/2020	767 Hangar Testing			
Inflight Tests	Airframe Section	Row/Seat Location	Gaspers	Mannequin Mask
Test 1	AFT	37A	OFF	OFF
Test 2	AFT	37A	OFF	OFF
Test 3	AFT	37A	OFF	OFF
Test 4	AFT	37B	OFF	OFF
Test 5	AFT	37B	OFF	OFF
Test 6	AFT	37B	OFF	OFF
Test 7	AFT	37D	OFF	OFF
Test 8	AFT	37D	OFF	OFF
Test 9	AFT	37D	OFF	OFF
Test 10	AFT	37E	OFF	OFF
Test 11	AFT	37E	OFF	OFF
Test 12	AFT	37E	OFF	OFF
Test 13	AFT	37F	OFF	OFF
Test 14	AFT	37F	OFF	OFF
Test 15	AFT	37F	OFF	OFF
Test 16	AFT	37K	OFF	OFF
Test 17	AFT	37K	OFF	OFF
Test 18	AFT	37K	OFF	OFF
Test 19	AFT	37L	OFF	OFF
Test 20	AFT	37L	OFF	OFF
Test 21	AFT	37L	OFF	OFF
Test 22	FWD	5A	OFF	OFF
Test 23	FWD	5A	OFF	OFF
Test 24	FWD	5A	OFF	OFF
Test 26	FWD	7A	OFF	OFF
Test 27	FWD	7A	OFF	OFF
Test 28	FWD	6D	OFF	OFF
Test 29	FWD	6D	OFF	OFF
Test 30	FWD	6D	OFF	OFF
Test 31	FWD	5L	OFF	OFF
Test 32	FWD	5L	OFF	OFF
Test 33	FWD	5L	OFF	OFF
Test 34	FWD-MID	18A	OFF	OFF
Test 35	FWD-MID	18A	OFF	OFF
Test 36	FWD-MID	18A	OFF	OFF
Test 37	FWD-MID	18B	OFF	OFF
Test 38	FWD-MID	18B	OFF	OFF
Test 39	FWD-MID	18B	OFF	OFF
Test 40	FWD-MID	18D	OFF	OFF
Test 41	FWD-MID	18D	OFF	OFF
Test 42	FWD-MID	18D	OFF	OFF
Test 43	FWD-MID	18E	OFF	OFF
Test 44	FWD-MID	18E	OFF	OFF
Test 45	FWD-MID	18E	OFF	OFF
Test 46	FWD-MID	18F	OFF	OFF
Test 47	FWD-MID	18F	OFF	OFF
Test 48	FWD-MID	18F	OFF	OFF
Test 49	FWD-MID	18K	OFF	OFF
Test 50	FWD-MID	18K	OFF	OFF
Test 51	FWD-MID	18K	OFF	OFF
Test 52	FWD-MID	18L	OFF	OFF
Test 53	FWD-MID	18L	OFF	OFF
Test 54	FWD-MID	18L	OFF	OFF
