# Supplementary material for: Aerosol tracer testing in Boeing 767 and 777 aircraft to simulate exposure potential of infectious aerosol such as SARS-CoV-2
Source: PLoS One. 2021 Dec 1;16(12):e0246916. doi: 10.1371/journal.pone.0246916 (PMC8635387; doi:10.1371/journal.pone.0246916)
Supplement: Attachment — Submitted filename: Questions to authors - Kinahan.docx [file pone.0246916.s017.docx]

Dear Authors,

Your manuscript is with no doubts of huge importance.

Prior sending it to external reviewers I would like to ask you to address few comments since I believe once you clarify these questions it should significantly help the review process.

Major Notes:

1) Page 6 L125-128:

Air exchange rates for the specific 767 and 777 airframes tested were reported to be 32 and 35 air changes per hour (ACH), respectively, with total cabin volumes of 9320 and 15075 cubic feet. Both ECS systems achieve approximately 50% of the air exchange through HEPA-filtered recirculation, and 50% through fresh air.

My comment:

It would be useful if you could provide more explanation on how the ventilation system works, and schematics of ventilation design would be very helpful. More details on IBAC and Airport MD8 aerosol sampler sampling port orientation are also needed. If sampling ports oriented horizontally and at the same time there is a strong vertical downward ventilation flow then it would be very helpful if you could provide linear velocities of the (assuming horizontal) aerosol sampling flow and of the downward ventilation flow. In case if ventilation downward flow linear velocity is significantly higher than horizontal aerosol sampling flow velocity then the aerosol sampling conditions are far from isokinetic and sampling efficiency could be low. If that’s the case, then the data presented in the manuscript may not necessarily reflect particles distribution across the plane. It may then rather represent actual (or close to actual) picture of how many particles could be inhaled by each passenger on the plane and this is of course very important, but it should be discussed and explained.

Thank you for taking the time to review, changes are also incorporated into the attached manuscript

Two diagrams below representing the 767 and 777 indicate the overall designed flow pattern. Designs attempt to minimize forward or aft flow, and flow should predominantly occur from top to bottom (downward), to help limit the spread of contaminants. The face velocity near the breathing zone is designed to operate between 10 and 60 ft/min (0.005-0.3 m/s) to limit the feeling of stagnant air or a breeze (National Research Council (U.S.). Committee on Airliner Cabin Air Quality. et al. 1986). At these low velocities and particle sizes, isokinetic inlets are unnecessary, and in the mixed environment unlikely to align perfectly. The IBAC samplers instead have omni-directional inlets, to sample from all directions immediately surrounding the inlet, located in the breathing zone. Similarly, the Airport MD8 samplers have large inlets facing forward into the breathing zone.

2) Pages 7,8: L171-174 Table 1 is confusing. The description says: “The effect of mask wearing during inflight testing and in an aerosol chamber. Top is the percent reduction in total counted particles for each release seat in triplicate with and without a mask. Bottom represents the aerosol chamber characterization for each tracer release, including total particles with and without a mask”.

- It is hard to understand from the description of the Top Table data (and from the Table itself), which data belong to with or without mask, and what CL stand for?
- Bottom Table shows I believe breathing with 1 micron fluorescent particles (both with mask and without mask) and 3 micron DNA tagged particles (perhaps without mask?).

Description of the Table 1 seems not accurate, please fix.

Thank you, this was two tables we were attempting to combine to reduce the total number, I have separated them into a table 1 and table 3 with better descriptions and more accurate location placement.

Minor Notes:

1) Environmental Control System (ECS) is deciphered only in Supplemental Materials (need to do it in the main text as well).

Understood, agreed, and fixed

2) Instantaneous Biological Analyzer and Collectors (IBAC, FLIR Systems) should be deciphered in 2.3 when it first appeared in the text.

Understood, agreed, and fixed
