# Supplementary material for: Aerosol tracer testing in Boeing 767 and 777 aircraft to simulate exposure potential of infectious aerosol such as SARS-CoV-2
Source: PLoS One. 2021 Dec 1;16(12):e0246916. doi: 10.1371/journal.pone.0246916 (PMC8635387; doi:10.1371/journal.pone.0246916)
Supplement: Attachment — Submitted filename: Review_PONE-D-21-03983.docx [file pone.0246916.s018.docx]

Review

Ms. Ref. No.: PONE-D-21-03983

Title: Aerosol tracer testing in Boeing 767 and 777 aircraft to simulate exposure potential of infectious aerosol such as SARS-CoV-2

Plos one

General comments:

This manuscript presents a study of aerosol release inside two aircrafts simulating potential exposure to SARS-CoV-2 viruses. The study is very topical and important. I recommend publication after my comments have been taken into account. These general comments are mainly my questions and opinions after reading the manuscript and any additions in these directions could help making the study more impactful. However, I let the authors decide how they can best respond to these comments.

Overall, this study is interesting, it is well designed and conducted, and provides important results concerning potential exposure to any air pollutants within an aircraft. As a first comment, I would like to see a critical disclaimer concerning the validity of this study in predicting the exposure to SARS-CoV-2 viruses. It is known from the literature that the size of SARS-CoV-2 virus is of the order of 120 nm. However, in some situations these viruses may exist as larger particles/droplets containing many viruses together with other material from sneezing, coughing, etc. Since the experiments of this study have been conducted using 1 and 3 μm particles, they do not exactly simulate the virus particles. In addition, the particle behaviour in an airflow and entering the sampler inlet is size dependent and behaviour of the two extremes – 120 nm and 3 μm – is very different and determined by different processes such as gravitational settling or inertia. These discussions could be added in the Discussion section to avoid too straightforward conclusions from these results to real life exposure.

It may be out of the scope of this study, but I remained curious to know whether the main results are something untypical in exposure scenarios or are these results similar to other potential exposure situations. In other words, it is expected that any point release of particles will be diluted as it enters the space in question and the question is if the reductions measured in this study indicate that the transport of viruses and consequently the exposure is insignificant. This study shows a minimum reduction of 99.54% of 1 μm aerosols from the index source to the breathing zone of a typical passenger seated directly next to the source. Would this value be similar or different in e.g. a restaurant situation with two people sitting next to each other?

Detailed comments:

The units: This is probably somewhat European perspective, but I find it always somewhat confusing that a mixed set of units are used. Naturally, it is the Journal policy, that determines the preferred unit system. I list here some of my observations:

-Line 126: The volume is given as cubic feet.

-Line 172: The particle concentration is given as “particles per liter of air”. Here the authors use different volume unit than previously. The abbreviation (pla) is unknown for me. This is used e.g. in line 200.

-Line 203: The instrument sampling rate is given as liters per minute (lpm).

-Line 236: The volume is given as milli liter, mL. Now the unit is written with capital L.

All of these are easy to understand, but perhaps some systematic way of using different units and symbols could be used.

Mask: I concluded that the mask was used for the mannequin that was the source of the particles. However, when reading the manuscript it was not immediately clear whether the source or the target was wearing the mask. This confusion is probably due to the fact that the discussion concerning the mannequin and the mask started already line 150, before the particle release process had been introduced (it comes rather late at chapter 2.8.).

The release process and the role of mannequin, chapter 2.4: The role of mannequin remained unclear. If I understand correctly, the aerosol release took place through the mannequin mouth/nose. However, the details remained unclear.

The size distributions, line 175 and supplemental fig 3: It is unclear what is presented in this figure. The y-axes unit for a size distribution is the size distribution function, not the concentration. The size distribution function is the concentration in given size bin normalized by the width of the bin, i.e. #/(cm3*(Dpmax-Dpmin)) which gives the unit for y-axes #/(cm3*μm). If the authors use the commercial instrument software this unit is probably the correct one automatically provided.

What is “ta” in line 179?

The sentence in lines 185-187 if difficult to understand.

The mask tests, chapter 3.2: What is the particle size used in these tests? If both particle sizes were used, was there any difference in the resulting reduction?
