# Supplementary material for: Aerosol tracer testing in Boeing 767 and 777 aircraft to simulate exposure potential of infectious aerosol such as SARS-CoV-2
Source: PLoS One. 2021 Dec 1;16(12):e0246916. doi: 10.1371/journal.pone.0246916 (PMC8635387; doi:10.1371/journal.pone.0246916)
Supplement: Attachment — Submitted filename: Manuscript Response to Reviewers Letter.docx [file pone.0246916.s019.docx]

Editorial Board

PLOS ONE

April 21, 2021

Editorial Board:

I am writing this letter to serve as a rebuttal to the revisions and points raised by the academic editor and reviewers for our manuscript entitled, “Aerosol tracer testing in Boeing 767 and 777 aircraft to simulate exposure potential of infectious aerosol such as SARS-CoV-2.” We believed the requested edits were reasonable, often necessary, and except in very limited circumstances did our best to accommodate them.

A point-by-point rebuttal appears below, as an appendix to this letter. We wanted to touch on the broader administrative and academic points brought up here as well. First, quantifying the number of releases has been cleaned up throughout the manuscript. Testing occurred over 8 days, but we have not investigated all of those releases to the level of detail presented here, numbers of tests for the 4 in-flight days analyzed here have been more clearly described, annotated, and reference the test tables in the supplement.

The particle size of the virus that most likely causes infection is probably not a single virus without sputum, cellular debris, salts, etc. Still, it places a good lower bound on potential particle size, and this has been more clearly specified, and what the implications are for Brownian motion vs gravitational settling and the test particles utilized here.

More details on the gaspers, the decision regarding whether to utilize them or not, the mannequin, and the IBAC sensor placement has been included to make clearer to the reader what the test team undoubtedly takes for granted.

With respect to the editorial notes two through four, an updated financial disclosure and competing interests statement appear in the page immediately following this letter. We have made every effort to fully disclose any financial or non-financial accommodation and overlap and have included the statements suggested by the editorial board.

Perhaps most importantly, to point five regarding the previous publication and overlap. The referenced document, which originally appears on US TRANSCOM’s website, was an initial analysis and look at the data provided by the authors to the US government sponsor. While the methods sections are nearly identical, the analysis is completely different, and much less detailed. The decision to make that internally provided document public by the government includes no copyright, no publisher, and notably, no peer-review process. We believe it should be more attune to a preprint server than a true publication for these reasons. Please let us know if you disagree and we shall try further distinguishing the remaining overlap.

I want to thank you again for your consideration and thoughtful comments. I’ve also attached an updated competing interests and funding statement immediately following this letter.

Sincerely,

Sean Kinahan
National Strategic Research Institute
University of Nebraska Medical Center
42nd and Emile St. Omaha, NE 68198
[skinahan@nsri.nebraskaresearch.gov](mailto:skinahan@nsri.nebraskaresearch.gov)
Tel: 1-443-255-3188

**Financial Disclosure:** These studies were sponsored by United States Transportation Command (USTRANSCOM) through an Army Research Office Contract (W911NF-17-C-0060) to Zeteo Tech Incorporated. Zeteo Tech provided project management, programmatic support, and technical expertise. Additional test plan review and expertise were provided by DARPA Biological Technologies Office (BTO) as a courtesy to USTRANSCOM. United Airlines won a competitive bid for initial testing and methods development, and then donated fuel, airplanes, and flight time for the follow-on testing presented here at no cost. Boeing answered engineering questions and provided operational feedback on ECS system performance at no cost as a courtesy to USTRANSCOM. Instruments were loaned by DHS Science and Technology Directorate via MIT Lincoln Laboratories, and the National Guard Bureau via L2 Defense. NSRI, S3I, and UNMC received subcontracting funding from Zeteo Tech to perform experiments and provide analysis and aerobiology expertise. The funder provided support in the form of salaries but did not have any additional role in the study design, data collection and analysis, decision to publish, or preparation of the manuscript. The specific roles of these authors are articulated in the ‘author contributions’ section.

**Competing Interests Statement:** S3I and Zeteo Tech Incorporated both operate in aerosol instrumentation and detection development, and their affiliation as private companies does not alter our adherence to PLOS ONE policies on sharing data and materials. No author has a previous or current competing interest with Boeing, United Airlines, or the data and instrumentation utilized herein.

**Point by Point Comment Rebuttal:**

**Reviewer #1:**

You used different tracers to study aerosol propagation in real aircrafts environments. But viruses in aerosols lose their biological activity at a rate that is a function of temperature, relative humidity, and other parameters. Therefore, the measurements carried out by the authors give only an upper estimate of the biological activity of the virus containing aerosol at the measurement points. This remark does not affect the conclusions drawn in the article about the low probability of passengers’ infection even in the nearest seats, however, I believe that the text of the article should contain a paragraph or two on the inactivation of SARS CoV-2 in aerosols, especially since information on the dependence of the inactivation rate of this virus on the above parameters are already presented in the literature.

We do not disagree with the point but hope 2-3 sentences is sufficient. We are keeping it short to stress that this study looks at the physics behind transport and exposure risk, not the biology and chemistry specific to this (or any) virus or biological. We are adding two citations and specifically mentioning their experimental conditions (UV light, Relative Humidity), and how any study on infection risk instead of exposure risk does need to consider biological and chemical activity or interactions.

Minor remarks
L48: there should be reference (2) instead of (1);
L563: please remove the journal's name after paper authors
Figs 2 and 3 are presented in the paper in very bad quality.

Authors agreed with all three minor remarks and have fixed each.

**Reviewer #2:**

1. Abstract. “We completed over 65 releases of 180,000,000 fluorescent particles …” This seems unnecessary and likely not that precise, e.g. 65 releases of 180M each or all together? At least change to exponential notation.

Agreed, changed to “over 65 releases of fluorescent tracer particles…”, to eliminate the number of particles.

2. Page 7. “2.2. 777-200 Testing. During the two days of in-flight testing, fluorescent tracer particles were released in the AFT, MID AFT, FWD, and FWD-MID sections of the airframe for a total of 64 releases. The breathing releases included 40 tests with the mannequin not wearing a mask and 24 tests with a mask.” This seems critical to understanding your methods/design and thus may need some clarification at this point. Fluorescent tracer particles nebulized? With what nebulizer? Were they fluorescent beads or solution? You give more specifics on these later in methods but some of the basic information would be helpful here.

Ordering multiple dependent methods was challenging, but added: “Method sections 2.4, 2.5, and 2.8 describe aqueous tracer solutions and nebulization methodology” to offer an intro into the methods and assure the reader that the how of the releases is addressed.

3. Page 8. “The test sizes were chosen…” The sizes you chose to test are supported by your references and appropriate for your sampling methodology, APS, for particles larger than 0.5um but some data also show that the size of the Covid-19 virus ranges from 75 to 200 nm (e.g. Chen N et al. Lancet 2020;395:507), outside the range of the APS (i.e. may need SMPS and/or CPC to count these smaller particles). It’s clear that you can’t test the full range of particle sizes that may contain virus but you should include the potential for smaller sizes in your discussion of limitations associated with your choices. The smallest airborne viruses would likely have different mobility than 1um particles, i.e. the former more diffusive.

Clarified the test aerosol is not the only particle size of potential importance. “Importantly, the physical size of SARS-CoV-2 has been reported as 50-200 nm (Chen, 2020), and while single virus particles as aerosols are unlikely, additional research defining the infectious particulate generated by patients is needed. As particle sizes become increasingly sub-micron the effects of gravity vs Brownian motion will shift, and physical transport will vary more from our larger test aerosols (Hinds, 1999).” In reality references are numbered and updated throughout document.

4. Page 11. “Standard surgical 3-ply masks …” What was type/manufacturer of mask? These may vary in terms of filtration efficiencies across manufacturers and fit to the face (especially a mannequin).

Company included, and a reclarification by using the term 3-ply disposable instead of surgical. Surgical has been used in media, but these brands are not truly medical masks at the consumer level. Specifically, “Standard disposable 3-ply masks (Guandong Paso Automible Technology Co., Ltd), which are the most likely to be handed out by an airline, were tested. A recent survey suggests that in the US, cloth masks were most commonly worn at least weekly by participants at 75%, but 3-ply masks were next most common at 57% of participants engaging in weekly use and this style is given out by airlines (33).”

5. Page 16 and Table 2. 767 has better filtration than 777 despite having lower ACH? Other differences to explain this?

Respectfully, this comment won’t be addressed specifically in the manuscript. There are many reasons that the larger plane may have worse removal in certain sections. Air mixing and supply to various parts of the cabin, variations in the design vs the functioning application in an older model, the seats or rows that happened to be tested, and seat spacing. Do not want to detract from the overall outcome and did not test specifically to determine the why.

6. Page 18 and table 3. “As further evidence of the mannequin’s mask redirecting particles rather than filtering them …”. Again this may be due to poor mask fitting for the specific mask used and the fact that it was on a mannequin as opposed to an actual face. Should state as limitation of this aspect of the study.

Agreed and added: Importantly, the fit of the mask to a mannequin face may vary from a human being, and increase noise if fit varies from location to location or test to test.

**Reviewer 3:**

comments
1. Page 2, line 30, Abstract. The 65 releases are hard to associate with the reported 85 releases for the 767 and 64 releases of the 777 listed on page 7, Methods.

Fixed the number of releases in the abstract, thank you. See more below.

2. Page 5, line 107. This sentence announces over 300 releases.

Clarified. We did over 300 releases over 8 days, but for length and time for analysis, only presenting the inflight data now, and only releases which represent breathing, the language has been improved to specify how many are studied here, and what the others represent, and why they were not yet analyzed due to time and word count.

3. Page 6, line 125. Were personal air vents on or off? My experience indicates most passengers usually have them on for varying flows. This can likely disturb flow from seat to seat or reduce particle count within the cone of air flow.

Added this to the manuscript, although personal gasper position was studied during ground testing, many aircraft do not have gaspers available, and in-flight testing conditions were limited. Other studies and existing modeling suggest their effect on infection risk is neutral (You, 2017). Consequentially, they remained in the off position for in-flight testing.

4. Page 7, line 59. 767-300 testing. Occasional power loss. When? How many times? During testing? Were tests repeated? Are these included in the 85 releases?

Clarified, the loss of total power was a ground testing phenomenon, not in the air, so is removed. The result of a loose electrical connection is the loss and non-inclusion of data from a specific seat, this is also indicated

5. Page 7, why are there 64 releases for 777 and 85 releases for 767? Specific test configurations/seating are difficult to determine: i.e., were there more than triplicate tests per seat or other tests? For instance, for the 767, there were 9 seats listed for triplicate, both mask and no mask tests. That adds up to 9 (seats)X3 (triplicate)X2 (masks)=54 tests. Yet 85 releases reported.

This has been clarified in the text, and a direct reference made to the test tables in the supplement, which contain every release location and condition.

6. Page 9, line 207. I am unfamiliar with IBACS. Were the IBAC sensor inlets located near typical face level and position?

Clarified that they are actively sampling via an omnidirectional inlet through a 1ft extension and into the instrument. These inlets are located in a breathing zone, or where an average persons face would reside when seated in flight.

7. Figure S2. I think I can only count 2 IBACS in the image although 4 is mentioned.

The other two are harder to see, and a different more camouflaged (darker grey) color. They have been marked in red bold in the updated supplement.

8. Could the volume taken up by actual persons in the seats change the results significantly? For instance, if a person takes up 25% of the volume in the sampled area, the counts would be the same, but concentration necessarily increases proportionally. And it may be important that concentration, in addition to counts, is important to infection.

We did consider this, the belief is that the difference is small, and causes an overestimation of how long particulate remains in the plane. A full plane will have a measurably smaller volume, with the same air flow, resulting in higher air exchanges. Will not be directly addressing in the text.

9. Page 20, line 448-454 Discussion should mention status of personal air vents.

As mentioned for the previous reviewer, this has now been addressed.

**Reviewer #4:**

Review

Overall, this study is interesting, it is well designed and conducted, and provides important results concerning potential exposure to any air pollutants within an aircraft. As a first comment, I would like to see a critical disclaimer concerning the validity of this study in predicting the exposure to SARS-CoV-2 viruses. It is known from the literature that the size of SARS-CoV-2 virus is of the order of 120 nm. However, in some situations these viruses may exist as larger particles/droplets containing many viruses together with other material from sneezing, coughing, etc. Since the experiments of this study have been conducted using 1 and 3 μm particles, they do not exactly simulate the virus particles. In addition, the particle behaviour in an airflow and entering the sampler inlet is size dependent and behaviour of the two extremes – 120 nm and 3 μm – is very different and determined by different processes such as gravitational settling or inertia. These discussions could be added in the Discussion section to avoid too straightforward conclusions from these results to real life exposure.

Understand the reviewer’s point and addressed both the uncertainty around typical COVID aerosol size, and the differing physics (increase in diffusion and Brownian motion) within the text. This was also mentioned by Reviewer 2.

It may be out of the scope of this study, but I remained curious to know whether the main results are something untypical in exposure scenarios or are these results similar to other potential exposure situations. In other words, it is expected that any point release of particles will be diluted as it enters the space in question and the question is if the reductions measured in this study indicate that the transport of viruses and consequently the exposure is insignificant. This study shows a minimum reduction of 99.54% of 1 μm aerosols from the index source to the breathing zone of a typical passenger seated directly next to the source. Would this value be similar or different in e.g. a restaurant situation with two people sitting next to each other?

We have studied other environments, that are difficult to incorporate here, and gone back and done ground testing with no ECS system operating. We will get this published as quick as we can. Basically, dilution helps an incredible amount, but is very location and airflow dependent. For example, we did a test in a large house, and it diluted into a large area, but then remained for longer period.

Detailed comments:

The units: This is probably somewhat European perspective, but I find it always somewhat confusing that a mixed set of units are used. Naturally, it is the Journal policy, that determines the preferred unit system. I list here some of my observations:

-Line 126: The volume is given as cubic feet.

Went ahead and changed this to cubic meters, thank you.

-Line 172: The particle concentration is given as “particles per liter of air”. Here the authors use different volume unit than previously. The abbreviation (pla) is unknown for me. This is used e.g. in line 200.

This is an aerosol term, ppl (particles per liter) or (#/l) have also been used in literature, but there is not a single accepted way of abbreviating aerosol concentration. They are also often expressed in per cubic meter and per cubic centimeter, depending on the instrumentation and environment.

-Line 203: The instrument sampling rate is given as liters per minute (lpm).

-Line 236: The volume is given as milli liter, mL. Now the unit is written with capital L.

All of these are easy to understand, but perhaps some systematic way of using different units and symbols could be used.

All changed to liters (lowercase l), thank you.

Mask: I concluded that the mask was used for the mannequin that was the source of the particles. However, when reading the manuscript it was not immediately clear whether the source or the target was wearing the mask. This confusion is probably due to the fact that the discussion concerning the mannequin and the mask started already line 150, before the particle release process had been introduced (it comes rather late at chapter 2.8.).

Briefly described the methodology in the chamber testing in 2.4 to better clarify.

The release process and the role of mannequin, chapter 2.4: The role of mannequin remained unclear. If I understand correctly, the aerosol release took place through the mannequin mouth/nose. However, the details remained unclear.

Added a clarification to the reason for the mannequin and where the output is plumbed in 2.8.

The size distributions, line 175 and supplemental fig 3: It is unclear what is presented in this figure. The y-axes unit for a size distribution is the size distribution function, not the concentration. The size distribution function is the concentration in given size bin normalized by the width of the bin, i.e. #/(cm3*(Dpmax-Dpmin)) which gives the unit for y-axes #/(cm3*μm). If the authors use the commercial instrument software this unit is probably the correct one automatically provided.

This is clarified now in the y-axis and the description. Yes, the data is normalized for bin width by taking the concentration and dividing by the bin width (dlogDp), because aerosol instruments often use different bin widths due to differing resolution on particle size.

What is “ta” in line 179?

Typo Fixed, thank you

The sentence in lines 185-187 if difficult to understand.

This was (in the original version, line numbers have naturally changed): For comparison, a four-mode fit for typical breathing nose to mouth fit diameters at 0.8 (86%), 1.8 (9%), 3. (3%) and 5.5 (2%) µm (25). We can see why, and it has been clarified.

The mask tests, chapter 3.2: What is the particle size used in these tests? If both particle sizes were used, was there any difference in the resulting reduction?

Clarified this was done with fluorescent tracer both above this section and in this section.

**Editor Comments:**

Those recommendations include: discussion on virus inactivation rate, quality of some of the figures, discussion on actual particle size that could contain virus in real-world (theoretically droplet containing single virus may dry down to ~100 nm but till it reached ~50 nm Brownian diffusion still should be very moderate therefore I would consider 1 micron size that you used for testing as appropriate but it should be discussed), mask fitting (mannequin vs real humans face) and more details on masks used (type, manufacturer), status of personal air-vents (In supplemental materials gaspers ON or OFF Columns are presented in the Tables, but there is not enough info about gaspers status in the main text. So, I would agree with this question and would like to have it more clearly written in the main text), location of IBAC sampling inlets (envisioning this question I've tried to clarify it with you prior to send manuscript to reviewers, but it seems like some more clarification is still needed).

Thank you for consideration, many edits have been made as specified above to better communicate these points.

Important question has been asked if your results are applicable to other environments (such as restaurants) and although this is of course out of scope of your work but recommendations on conducting similar studies in other populated environments could be discussed.

I touch on this briefly in the introduction to the IBAC. This type of work has been done before, but not enough for infectious disease. It is typically utilized for testing filtration, for biodefense, or in hospital environments. We would love to study alternative environments. One nice thing about airplanes is there are limited models and designs, whereas restaurants have much more varied airflows and layouts.

There are other minor comments/questions that you can find while reading each reviewer feedback. So, I would like to request these minor revisions and hope after these revisions been made we can proceed with publication.

Thank you again for your consideration.
