# Supplementary material for: Aerosol tracer testing in Boeing 767 and 777 aircraft to simulate exposure potential of infectious aerosol such as SARS-CoV-2
Source: PLoS One. 2021 Dec 1;16(12):e0246916. doi: 10.1371/journal.pone.0246916 (PMC8635387; doi:10.1371/journal.pone.0246916)
Supplement: Attachment — Submitted filename: Academic Editor Notes to revised version.docx [file pone.0246916.s020.docx]

Dear Authors,

Thank you for revising the manuscript and addressing reviewer’s comments.

I think your manuscript is now in a much better shape but just a few minor edits/corrections are still required, please see below:

Line numbers were used from the “Revised Manuscript with Track Changes” version:

L52: “While large-droplet transmission likely does not vary substantially from the unique circumstance of being on an aircraft, the aerosol exposure route is dependent on the ventilation, environment”

*Indicate please size of “large droplets”.*

L160: tests with a mask. Methods sections 2.4, 2.5, and 2.8 describe aqueous tracer solutions and

*You don’t have these 2.4, 2.5, and 2.8 numbering anymore.*

L180: “minutes, and then placed into a static mode without airflow. The test solution, which is a 10% dilution of the stock solution in deionized water, was nebulized identically to in-flight tests, from a nebulizer plumbed to a mannequin (fluorescent tracer) with or without a mask, or without a mannequin (DNA-tagged tracer) and then briefly mixed using two remote-controllable fans (20-25 seconds).”

*What was the stock solution?*

*Fluorescent tagged microspheres were aerosolized for one minute in a breathing pattern for 2 seconds on and 2 seconds off, correct?*

L190: “for each release condition has low standard error around the mean, with a total number of fluorescent tracers when unmasked of 1.8*10^8^, compared to 2.4*10^7^ beads for the larger 3 µm DNA-tagged tracers (Table 1)”.

*In the Table 1 you have both with and without mask results for fluorescent tracers but not in the text. I would suggest to add results for with masks (in the brackets): “for each release condition has low standard error around the mean, with a total number of fluorescent tracers when unmasked of 1.8*10^8^ (with mask 1.7*10^8^) compared to 2.4*10^7^ beads for the larger 3 µm DNA-tagged tracers (Table 1)”.*

L206: “sizes become increasingly sub-micron the effects of gravity vs Brownian motion”

*For submicron particles its not a gravitational force – its inertial force. So I would change it to:*

*“sizes become increasingly sub-micron the effects of inertial forces vs Brownian motion”*

L229: “IBAC sensors were placed in individual seats, with either 1 foot tubing extensions to actively sample within the breathing zone or placing on a collapsible crate to achieve the same sampling location”

*How long was the sampling time?*

L235: “for isokinetic sizing and alignment with a flow field”

*Isokinetic sampling (not sizing)*

L240: “typical resting passenger minute volume (7.5 lpm) rather than the instruments sampling rate of”

*I think you need to clarify respiratory minute volume*

L263: “(Sartorius), which operated for fifteen minutes, and collects 99.9995% of particles”

*Need to specify instrument specs: what particle size range it collects*

L265: “Five high volume air collectors were distributed near release”

*Are those the same Airport MD8 or different air-samplers?*

L289: “minutes and fluorescent tagged microspheres were generated aerosolized for one minute in a breathing pattern for 2 seconds on and 2 seconds off”

*That is for both in the Chamber and on the plane, correct?*

L316: “Economy (with and without a mask) and first class penetration percentages for nearby seats on the”

*Although you previously explained very clear that terms “with and without a mask” relate to mannequin release of particles, some readers might still be confused and may think that here you are talking about mask protection for inhalation. I understand that for attentive reader it should be clear that you didn’t put mask on IBAC😊 but some people may still think that you somehow assessed mask inhalation protection. To avoid this potential confusion, I would suggest something like: “Economy (mannequin particles release with and without a mask)”. And for the Table 2 instead of “Breathing w/o mask or w/mask” I would also recommend to use “mannequin particles release with and without a mask”. Same comment for Fig 2 Caption: term “unmasked breathing” might be confusing.*

Supplemental Tables 7 and 8 include “coughing” test conditions – What does it mean? I don’t think there is any mentioning about testing “coughing conditions” in the text.

Once again: What I didn’t find is how long was the IBAC sampling after each release. 15 min (same as Airport MD8 aerosol samplers)?

I also think that short discussion about how your data correspond to the real-world conditions is required. Please see below some thoughts:

According to Asadi 2020^1^: “certain individuals are “speech superemitters” who emit an order of magnitude more aerosol particles than average, about 10 particles/second. A ten-minute conversation with an infected, asymptomatic superemitter talking in a normal volume thus would yield an invisible “cloud” of approximately 6,000 aerosol particles that could potentially be inhaled by the susceptible conversational partner or others in close proximity”. Assuming this is an approximation of upper limit we may estimate that during one minute talk this supermitter will release ~1,000 particles. It means that according to your measurements ~0.5% could reach the breathing zone of a typical passenger seated directly next to the source, or ~5 particles (for one minute release). For all other passengers it will be 0.02 - 0.03 % or 0.2 – 0.3 particles. Although infectious dose for COVID-19 is not known (at least I am not aware if any published literature data have this info), it might be as low as one particle. Therefore, sitting next to superemitter might not be safe.

1.	Asadi, S., et al., *The coronavirus pandemic and aerosols: Does COVID-19 transmit via expiratory particles?* 2020, Taylor & Francis.
