# Supplementary material for: Aerosol tracer testing in Boeing 767 and 777 aircraft to simulate exposure potential of infectious aerosol such as SARS-CoV-2
Source: PLoS One. 2021 Dec 1;16(12):e0246916. doi: 10.1371/journal.pone.0246916 (PMC8635387; doi:10.1371/journal.pone.0246916)
Supplement: Attachment — Submitted filename: Response to Reviewers.docx [file pone.0246916.s021.docx]

Editorial Board

PLOS ONE

October 1, 2021

Editorial Board:

I am writing this letter to address the questions and revisions raised by the academic editor and reviewers for our most recent version of the manuscript entitled, “Aerosol tracer testing in Boeing 767 and 777 aircraft to simulate exposure potential of infectious aerosol such as SARS-CoV-2.”

A point-by-point rebuttal appears below, as an appendix, as we did during the last revision. Overall, we attempted to accept, revise, and clarify at each point brought up in the review, and have responded below in blue ink. These changes were all relatively minor but important. We have also updated references that were preprints to their peer-reviewed final versions and fixed some non-Vancouver formatting.

Thank you for your time and consideration.

Sincerely,

Sean Kinahan
National Strategic Research Institute
University of Nebraska Medical Center
42nd and Emile St. Omaha, NE 68198
[skinahan@nsri.nebraskaresearch.gov](mailto:skinahan@nsri.nebraskaresearch.gov)
Tel: 1-443-255-3188

**Point by Point Comment Rebuttal:**

I think your manuscript is now in a much better shape but just a few minor edits/corrections are still required, please see below:

Line numbers were used from the “Revised Manuscript with Track Changes” version:

L52: “While large-droplet transmission likely does not vary substantially from the unique circumstance of being on an aircraft, the aerosol exposure route is dependent on the ventilation, environment”

*Indicate please size of “large droplets”.*

To avoid a specific size distinguishing between aerosols and droplets (typically between 20-100+ microns), best suited for other publications, we have changed the wording to “projectile droplet transmission, dominated by their own inertia” and included two new references that do cover this topic in more detail. The point here is that we are looking at aerosols and not the effect of someone sneezing in a Newtonian physics way that would directly transfer a virus-laden droplet from the mouth or nose to the mucus membrane of someone nearby.

L160: tests with a mask. Methods sections 2.4, 2.5, and 2.8 describe aqueous tracer solutions and

*You don’t have these 2.4, 2.5, and 2.8 numbering anymore.*

Thank you, this was changed to “described below.” Since this is the first paper in such detail about these methods across multiple sections and subsystems, we wanted to ensure readers that the methods are covered.

L180: “minutes, and then placed into a static mode without airflow. The test solution, which is a 10% dilution of the stock solution in deionized water, was nebulized identically to in-flight tests, from a nebulizer plumbed to a mannequin (fluorescent tracer) with or without a mask, or without a mannequin (DNA-tagged tracer) and then briefly mixed using two remote-controllable fans (20-25 seconds).”

*What was the stock solution?*

*Fluorescent tagged microspheres were aerosolized for one minute in a breathing pattern for 2 seconds on and 2 seconds off, correct?*

This was reworded to get rid of the 10% dilution, instead describing the specific numbers of beads. The nebulization settings being identical to inflight (yes, 2 s on and 2 s off), is also clarified by describing it as identical to in-flight tests.

L190: “for each release condition has low standard error around the mean, with a total number of fluorescent tracers when unmasked of 1.8*10^8^, compared to 2.4*10^7^ beads for the larger 3 µm DNA-tagged tracers (Table 1)”.

*In the Table 1 you have both with and without mask results for fluorescent tracers but not in the text. I would suggest to add results for with masks (in the brackets): “for each release condition has low standard error around the mean, with a total number of fluorescent tracers when unmasked of 1.8*10^8^* *(with mask 1.7*10^8^) compared to 2.4*10^7^ beads for the larger 3 µm DNA-tagged tracers (Table 1)”.*

Agree with this change and incorporated into the text.

L206: “sizes become increasingly sub-micron the effects of gravity vs Brownian motion”

*For submicron particles its not a gravitational force – its inertial force. So I would change it to:*

*“sizes become increasingly sub-micron the effects of inertial forces vs Brownian motion”*

Agree with this change, we were attempting to describe the difference between our test aerosols and a potentially much smaller aerosol, but it was worded poorly, thank you.

L229: “IBAC sensors were placed in individual seats, with either 1 foot tubing extensions to actively sample within the breathing zone or placing on a collapsible crate to achieve the same sampling location”

*How long was the sampling time?*

Substantial text added to clarify that instruments sample at 1-second intervals, and that the advantage of real-time, is that sampling time was based on a return to baseline, which is of course unique for any testing environment. The team monitors a fraction (approximately 10), but we did a few more the first day, to make sure we had a representative sample returning to baseline. Monitoring in real-time is more challenging and requires ethernet cables, so its not possible to do all 40+ sensors this way.

L235: “for isokinetic sizing and alignment with a flow field”

*Isokinetic sampling (not sizing)*

Agree with this change, sizing was the incorrect word to use.

L240: “typical resting passenger minute volume (7.5 lpm) rather than the instruments sampling rate of”

*I think you need to clarify respiratory minute volume*

Agree with this change and incorporated.

L263: “(Sartorius), which operated for fifteen minutes, and collects 99.9995% of particles”

*Need to specify instrument specs: what particle size range it collects*

I reread the paper, added another citation, and reduced the claim. While that paper does indeed claim 99.9995%, another paper with a similar methodology was 96%. Both citations are now included. Additionally, the test sizes (0.7 to 1.0 micron) and particle type (*Bacillus* spores) are included.

L265: “Five high volume air collectors were distributed near release”

*Are those the same Airport MD8 or different air-samplers?*

Yes, they are the same collector, changed to Five of these air collectors instead of five high-volume air collectors.

L289: “minutes and fluorescent tagged microspheres were generated aerosolized for one minute in a breathing pattern for 2 seconds on and 2 seconds off”

*That is for both in the Chamber and on the plane, correct?*

Yes, thank you, this is clarified this in the chamber description above.

L316: “Economy (with and without a mask) and first class penetration percentages for nearby seats on the”

*Although you previously explained very clear that terms “with and without a mask” relate to mannequin release of particles, some readers might still be confused and may think that here you are talking about mask protection for inhalation. I understand that for attentive reader it should be clear that you didn’t put mask on IBAC😊 but some people may still think that you somehow assessed mask inhalation protection. To avoid this potential confusion, I would suggest something like: “Economy (mannequin particles release with and without a mask)”. And for the Table 2 instead of “Breathing w/o mask or w/mask” I would also recommend to use “mannequin particles release with and without a mask”. Same comment for Fig 2 Caption: term “unmasked breathing” might be confusing.*

Agree to these changes to make sure that readers understand mask use was solely tested on the source. It is our belief that the inhalational side can be addressed by readers or modelers by applying a variety of other data sets that have examined mask efficiency. Specifically, used the phrasing: (particles generated through the mannequin with and without a mask.

Supplemental Tables 7 and 8 include “coughing” test conditions – What does it mean? I don’t think there is any mentioning about testing “coughing conditions” in the text.

Have changed lines 120 and 121 to discuss the data on figshare to say: “including limited seats and releases tested at an accelerated release velocity, not analyzed here, to more closely mimic coughing (Supplement)” The amount of data and words in the article were already long. We have started a second manuscript looking at data of coughing, people walking, and bathroom use. If you are curious, it behaved similarly to the breathing simulation, except more of the aerosol went forward first. More interestingly but not unexpectedly, the mask captures substantially more initial material when it is traveling at a much faster velocity.

Once again: What I didn’t find is how long was the IBAC sampling after each release. 15 min (same as Airport MD8 aerosol samplers)?

Thank you, please see above. This was a shorter duration than the Airport MD8, because they could be monitored in real-time. It was essentially always less than 8 minutes, often closer to 6, but dependent on location and spacing. If one downloads the real-time data or sheets available on Figshare, the start/stop times of all tests are there, and they can see the exponential decay back to a baseline level. Still, we believe the description of return to baseline in <8 minutes (as described above) is sufficient.

I also think that short discussion about how your data correspond to the real-world conditions is required. Please see below some thoughts:

According to Asadi 2020^1^: “certain individuals are “speech superemitters” who emit an order of magnitude more aerosol particles than average, about 10 particles/second. A ten-minute conversation with an infected, asymptomatic superemitter talking in a normal volume thus would yield an invisible “cloud” of approximately 6,000 aerosol particles that could potentially be inhaled by the susceptible conversational partner or others in close proximity”. Assuming this is an approximation of upper limit we may estimate that during one minute talk this supermitter will release ~1,000 particles. It means that according to your measurements ~0.5% could reach the breathing zone of a typical passenger seated directly next to the source, or ~5 particles (for one minute release). For all other passengers it will be 0.02 - 0.03 % or 0.2 – 0.3 particles. Although infectious dose for COVID-19 is not known (at least I am not aware if any published literature data have this info), it might be as low as one particle. Also quantifying infectious pathogen loads in exhaled air is technically challenging, so it is hard to predict what percentage of particles exhaled by super-emitter contains pathogens. Assuming that every exhaled particle contains pathogen (and I don’t know how valid this assumption is), sitting next to superemitter might not be safe.

1.	Asadi, S., et al., *The coronavirus pandemic and aerosols: Does COVID-19 transmit via expiratory particles?* 2020, Taylor & Francis.

Thank you, a discussion paragraph was added (reproduced below) to sum up our belief that the data demonstrates overall risk of superspreader events throughout the plane is limited, but for a given passenger is dependent on distance from a source. Specifically mentioning this paper and one other on superemitters generating 10x+ more aerosols, and using the example of a single infectious dose in a single particle. These closest seats are also where large ballistic droplets and changes in orientation of the source have the largest effect.

The infectious dose and viral load emitted by an infected passenger may also depend on their disease severity, length of infection, age and profile, many variables which are currently unknown. As an example, the number of particles emitted may vary substantially, with superemitters generating an order of magnitude or more aerosols than a typical person while talking or breathing, perhaps as many as 6000 in ten minutes of conversation (46, 47). While the percentage of these that contain infectious material, and the infectious dose are still unknown, the purpose of this analysis is to demonstrate the risk to most passengers is low and decreases rapidly with distance. However, being seated next to shedding passenger likely still carries risk, especially depending on assumptions regarding these unknown variables. If a single particle contains an infectious dose, low penetration percentages would still be sufficient for those closest passengers to a source.
